# Assessment of Audio‐Visual and Print Educational Material using PEMAT for Prevention of Dementia: A SMRUTHI, India Initiative

**DOI:** 10.1002/alz70861_109017

**Published:** 2025-12-23

**Authors:** Kritika Sharma, Anu Gupta, Nilima N, Hina Narzari, Shubham Gupta, Aditi Dubey, Varuna Sharma, Sakshi Sharma, Kajal Fulara, Sneha, Kaamini Kashyap, Shikha Chaudhary, Krishan Kaushal, Phaniraj Vastrad, Priya darshanraj N, Sandip Bhattacharjee, Gajraj Singh Shekhawat, Harshath Ajay V, Sandesh J R, Ambika YV, Bishakha Sen, Shalina Jamatia, Priyanka Kumari Meena, Hitesh Tiwari, Shaily Bhushan, Sagar PM, Ripanjit Singh, Manish Acharjee, Kalpna Sharma, Aishwarya Bhovi, Mailarappa Padiyappa Hooli, Shilpa K, Naveen MR, Dipankar Deb, Pameli Jamatia, Pooja Sharma, Akhilesh Nagar, Tinku Yogi, Rishav Bhatia, Rishabh Gautam, MA Khan, Vaibhav Patil, Rajeev Aggarwal, Ashima Nehra, Subarna Roy, Manish Barvaliya, Subrata Baidya, Shampa Das, P K Anand, Sunil K Raina, Abhik Sinha, Venugopalan Y Vishnu, MV Padma Srivastava

**Affiliations:** ^1^ All India Institute of Medical Sciences, Delhi, Delhi India; ^2^ All India Institute of Medical Sciences, New Delhi, Delhi India; ^3^ All India Institute of Medical Sciences, New Delhi India; ^4^ All India Institute of Medical Sciences, New delhi, Delhi India; ^5^ MRHRU, Raichur, Karnataka India; ^6^ MRHRU, Khumulwmg, Tripura India; ^7^ Model Rural Health Research Unit (MRHRU), Jaipur, Rajasthan India; ^8^ Model Rural Health Research Unit (MRHRU), Sirwar, Raichur India; ^9^ Model Rural Health Research Unit (MRHRU), Sirwar, Raichur, Karnataka, India, Karnataka India; ^10^ Model Rural Health Research Unit (MRHRU), Khumulwng, Tripura India; ^11^ MRHRU, Jaipur, Rajasthan India; ^12^ Department of Community Medicine, Dr. RP Government Medical College, Tanda, Himachal Pradesh, India, Tanda, Himachal Pradesh India; ^13^ MRHRU, Sirwar, Karnataka India; ^14^ f) Department of Community Medicine, Dr. RP Government Medical College, Tanda, Himachal Pradesh, India, Una, Himachal Pradesh India; ^15^ Model Rural Health Research Unit (MRHRU), Raichur, Karnataka India; ^16^ MRHRU, Una, Himachal Pradesh India; ^17^ ICMR‐NITM, Belagavi, MRHRU Sirwar, Raichur, Karnataka India; ^18^ In charge MRHRU Khumulwmg, Tripura India; ^19^ Community Medicine AGMC, Khumulwng, Tripura India; ^20^ ICMR‐NIIRNCD, Jodhpur & Nodal Officer, MRHRU Bhanpur Kalan, Jaipur India; ^21^ Department of Community Medicine, Dr. RP Government Medical College, Tanda, Himachal Pradesh India; ^22^ ICMR Center for Ageing and Mental Health (CAMH), Kolkata India

## Abstract

**Background:**

SMRUTHI‐India is a multimodal initiative to establish a cohort across four sites in India for randomized controlled interventions to prevent dementia in the at‐risk elderly population. Dementia prevalence in India is 7.9%, with higher rates in rural than urban areas. Given the high prevalence, low health literacy, and multicultural context, developing relevant print and audiovisual materials for psycho‐education tailored to rural populations is essential.

**Method:**

The Care Bundle Module (CBM) was developed by a multidisciplinary team at AIIMS, New Delhi focused on the prevention of Dementia in rural population. The CBM Booklet was created and Content Validity Index (CVI) was also calculated based on feedback from five experts. It was then transformed into 10 animated videos in three stages: Pre‐Production, Production and Post‐Production. To evaluate “understandability” and “actionability” of both the booklet and videos, the Patient Educational Material Assessment Tool (PEMAT) for print (P) and audio‐visual (AV) formats was used among 83 (P) and 86 (AV) participants across the sites. Qualitative feedback from the target population and a behavioral expert was also taken and is currently being incorporated into the material.

**Result:**

The CBM Booklet’s S‐CVI/Avg was 0.933, indicating 93.3% content validity based on expert ratings. PEMAT understandability scores averaged 96.35% (Print) and 95.46% (Audio‐Visual), while actionability scores were 97.90% (P) and 98.75% (AV). PEMAT‐based CVI showed strong regional agreement: Himachal Pradesh reported S‐CVI/Avg of 0.9802 (P) and 0.993 (AV), Kappa 0.98 and 0.993; Karnataka 0.943 (P) and 0.975 (AV), Kappa 0.94 and 0.97; Rajasthan 0.9828 (P) and 0.9791 (AV), Kappa 0.9829 and 0.979; Tripura 0.9921 (P) and 0.9053 (AV), Kappa 0.9921 and 0.902. These results show high I‐CVI and Kappa across regions, confirming excellent content validity and inter‐rater agreement.

**Conclusion:**

The CBM materials demonstrated strong agreement in both experts and target population as reflected in high S‐CVI/Avg and Kappa values across regions. PEMAT scores confirmed excellent understandability and actionability in both print and audiovisual formats. These results highlight the relevance and effectiveness of the CBM in delivering dementia‐related psychoeducation in rural India.

